# Correction to ‘How to build a new athletic track to break records’

**DOI:** 10.1098/rsos.200888

**Published:** 2020-06-03

**Authors:** Amandine Aftalion, Emmanuel Trélat

*R. Soc. Open Sci.* 7, 200007. (Published 25 March 2020). (doi:10.1098/rsos.200007)

This correction refers to an error in the caption of Figure 3. A radius of ‘44.53 m’ was given in place of ‘44.88 m’. This has now been corrected.

